# The Challenging Diagnosis of Interstitial Lung Disease in Children—One Case Report and Literature Review

**DOI:** 10.3390/jcm11226736

**Published:** 2022-11-14

**Authors:** Marcela Daniela Ionescu, Nicoleta Aurelia Popescu, Diana Stănescu, Augustina Enculescu, Mihaela Bălgrădean, Georgiana Mihaela Căpitănescu, Dragos Bumbăcea

**Affiliations:** 1 Department of Pediatrics, “Carol Davila” University of Medicine and Pharmacy, 020021 Bucharest, Romania; 2 “Marie S. Curie” Emergency Children’s Clinical Hospital, 041451 Bucharest, Romania; 3 Department of Cardio-Thoracic Medicine, “Carol Davila” University of Medicine and Pharmacy, 020021 Bucharest, Romania; 4 Department of Pneumology and Acute Respiratory Care, Elias Emergency University Hospital, 041451 Bucharest, Romania

**Keywords:** interstitial lung disease, hypersensitivity pneumonitis, children, avian antigen, bronchoalveolar lavage

## Abstract

Childhood interstitial lung disease (chILD) includes a heterogeneous spectrum of rare respiratory disorders in children associated with substantial morbi-mortality. Interstitial tissue, and other pulmonary structures, epithelium, blood vessels, or pleura are involved, resulting in a restrictive lung disfunction. Respiratory symptoms set in progressively and are often subtle, making thorough clinical history and physical examination fundamental. The etiology often is obscure. The clinical presentation mimics pneumonia or asthma, leading to a diagnostic delay. Challenging diagnosis may require genetic tests, bronchoalveolar lavage, or lung biopsy. Alongside general supportive therapeutic measures, anti-inflammatory, immunosuppressive or antifibrotic agents may be used, based on data derived from adult studies. However, if accurate diagnosis and treatment are delayed, irreversible chronic respiratory failure may ensue, impacting prognosis. The most frequent chILD is hypersensitivity pneumonitis (HP), although it is rare in children. HP is associated with exposure to an environmental antigen, resulting in inflammation of the airways. Detailed antigen exposure history and identification of the inciting trigger are the cornerstones of diagnostic. This article provides the current state of chILD, revealing specific features of HP, based on a clinical case report of a patient admitted in our clinic, requiring extensive investigations for diagnosis, with a favorable long-term outcome.

## 1. Introduction

Childhood interstitial lung disease (chILD) comprises a broad spectrum of rare respiratory disorders in children, with important differences compared with adults, including immunological and developmental abnormalities. The causes of this disorder often remain unknown. Information on chILD global epidemiology remains extremely limited because of a lack of systematic registries to collect these cases. Incidence estimates varied from 0.13 to 16.2 cases/100,000 children < 17 years of age/year, these figures underestimating the actual number of cases, considering the lack of a standardized definition system, and collected data being reported to small series of patients [1,2]. The interstitial tissue, as well as other pulmonary parenchymal components such as epithelium, airways, vessels, or pleura, may be involved. Thus, the spectrum of chILD is very large. Restrictive lung physiology with significant impairment of gas exchange develops, associating important morbidity and mortality [3,4].

Hypersensitivity pneumonitis (HP), also called extrinsic allergic alveolitis, is a form of immune-mediated inflammatory interstitial lung disease involving the distal respiratory airways, alveoli, and pulmonary interstitial tissue, which results from repeated exposure to a variety of inhaled environmental organic antigens. Being associated with the occupational setting, it is relatively uncommon in pediatric patients. It accounts for approximately 50 percent of all forms of interstitial lung disease in this age group of patients, but its real pediatric incidence is usually underestimated because of underdiagnosis [5,6]. Between the years 1960 and 2005, 95 cases of HP were reported in children [7].

The purpose of this article is to provide the current state of interstitial lung disease in children, revealing specific features of HP in this age group, underlined by a clinical case report of a child admitted to the Pediatric Department of the “Marie S. Curie” Emergency Children’s Clinical Hospital, Bucharest, Romania.

## 2. Literature Review

### 2.1. Classification of Interstitial Lung Disease in Infants and Children

The diagnosis of chILD is most frequent in the first year of life, and more than 10 percent of cases are familial [3]. In older children, the pathogenesis of chILD is similar to adults’ processes. However, there are major differences in etiology, natural history, prognosis, and management between children and adults, and the classification of interstitial lung disease in adults underestimates chILD [8,9]. The most widely accepted classification of chILD was published in 2007 by the Children’s Interstitial Lung Disease Research Cooperative. In 2013, this was included in the American Thoracic Society clinical practice guidelines [10]. The classification system is based on clinical and histopathological characteristics. Age at presentation is a key factor, dividing chILD into that more common in infancy (under two years of age) and that not specific to infancy (over two years of age). A third group of unclassifiable entities is also described [10,11].

The chILD more common in infancy is further divided into four main categories, and each of these entities is subdivided into multiple pathologic conditions: diffuse developmental disorders (acinar/alveolar dysgenesis, congenital alveolar dysplasia); lung growth abnormalities (pulmonary hypoplasia, chronic neonatal lung disease, for instance due to prematurity, structural changes due to chromosomal anomalies or associated with congenital heart disease); surfactant dysfunction disorders; and other specific conditions of unknown or poorly understood etiology (neuroendocrine cell hyperplasia of infancy, pulmonary interstitial glycogenosis) [10,11,12].

In older children, the classification system of chILD, according to history and clinical presentation, includes the host immune status (disorders of the immunocompetent or immunocompromised host), the presence of a systemic disease process (immune-mediated disorders, pulmonary hemorrhage syndromes, collagen vascular disease, sarcoidosis, malignant infiltrates) and the association of a vascular disorder masquerading as interstitial lung disease (arterial hypertensive vasculopathy, pulmonary edema, thromboembolic disease, lymphatic disorders). The main causes of chILD in a normal host are related to infectious and post-infectious processes, exposure to environmental agents (HP), aspiration syndromes, and eosinophilic pneumonia. Interstitial lung disease etiology in immune-compromised hosts includes opportunistic infections, disorders related to therapeutic intervention or transplantation, and lymphoid infiltrates [2,10,12].

### 2.2. Clinical Presentation of chILD

The clinical manifestations are often subtle or nonspecific. They vary from an asymptomatic presentation with accidental radiologic features suggestive of interstitial lung disease, to typical characteristic respiratory symptoms such as dyspnea, tachypnea, cough, exercise intolerance, tiring during feeding, respiratory infections, and hemoptysis. The onset of chILD is usually insidious, and the age of the patient is a key factor [2,13,14]. 

The physical examination of chILD is frequently abnormal, but the findings are nonspecific. Tachypnea, retractions, and basal inspiratory crackles are typically present. Chronic dry cough, cyanosis, clubbing, failure to thrive, and loud second heart sound due to pulmonary hypertension can be related, suggesting advanced disease [15]. Extrapulmonary signs and symptoms may help narrow the differential diagnosis, particularly in older children. These may include joint disease, cutaneous rashes, musculoskeletal manifestations, lymphadenopathy, hepatosplenomegaly, suggesting pulmonary disease in the context of a systemic disorder [16,17].

### 2.3. Diagnosis Approach of chILD

A systematic approach to the diagnosis of chILD is essential and it should begin with preparing a careful clinical history and performing a detailed physical examination, followed by non-invasive (physiological and laboratory investigations, chest radiography, high-resolution computed tomography scan, and echocardiography) and invasive (bronchoalveolar lavage and lung biopsy) investigations [2]. Details should be obtained on the history of prematurity, cardiac disease, chromosomal anomalies, family history, and on precipitating factors (severe respiratory infections, environmental exposure to organic or inorganic agents, and use of medication with pulmonary toxicity) [3,18]. 

After excluding the most common known causes of lung disease, such as pulmonary infections, recurrent aspiration, structural airway abnormalities, bronchopulmonary dysplasia, congenital heart disease, immunodeficiencies, and cystic fibrosis, a chILD syndrome may be considered if the child meets at least three of the criteria presented in Table 1 [10].

Once the chILD syndrome is established, further non-invasive investigations are performed. 

Laboratory tests rarely establish the diagnosis, but they may be helpful to exclude other systemic disorders that can be associated with interstitial lung disease. These can be grouped into immune function tests, autoantibodies studies, infection and inflammation markers, tests specific for environmental antigen exposure, and other non-specific analyses. Genetic tests are now available, allowing for the non-invasive diagnosis of a growing number of genetic etiologies in chILD [2].

Pulmonary function tests and pulse oximetry are used for the assessment of severity in chILD [3]. Spirometry, lung volumes, and diffusing capacity should always be obtained, depending upon the child’s cooperation. Interstitial lung disease is usually characterized by restrictive rather than obstructive pulmonary dysfunction, with reduced lung compliance and decreased lung volumes, together with a reduction in diffusing capacity of the lungs for carbon monoxide (DLCO) [19,20]. Oxygen saturation is normal with mild disease, but progressive illness determines desaturation during sleep or exercise, and, further, hypoxemia at rest. Hypercarbia appears only late in the disease course [3,21].

Imagistic techniques are necessary for the assessment of chILD. Chest radiography is usually the preferred initial imaging study. Frequently, it shows pulmonary abnormalities, with interstitial, alveolar, or mixed infiltrates, and hyperinflation. However, it rarely provides a specific diagnosis and, also, it may appear normal in early or mild forms of chILD [22,23]. Thus, further imaging characterization using High-Resolution Computer Tomography (HRCT) is necessary. Different lesions may be described, such as septal thickening, honeycombing aspect, ground-glass opacification, mosaic attenuation, lung cysts, or nodules. In advanced forms of the disease, fibrotic lesions can be observed [3,24]. Unique features to some diseases may also be revealed, reducing the need for lung biopsy [3,25].

Evaluation for pulmonary hypertension should be considered early because of the significant impact on the prognosis. Echocardiography is usually preferred, and it should be performed to estimate pulmonary artery pressure and exclude cardiac defects. In rare situations, cardiac catheterization is necessary for a more accurate evaluation [10,26].

**Figure 1:**
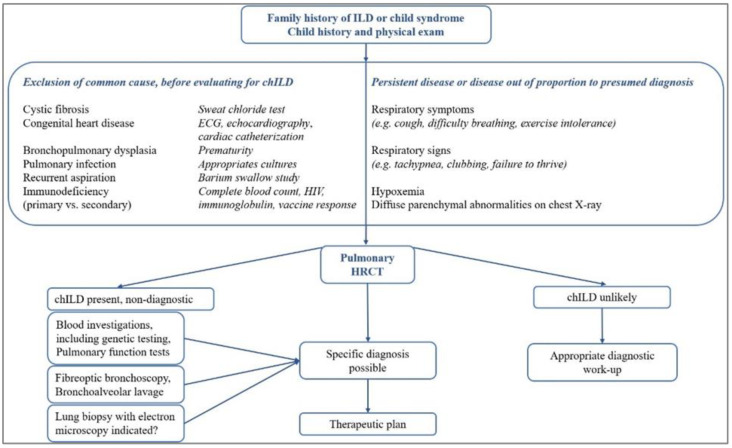
General diagnosis design in chILD.

If the diagnosis cannot be made using non-invasive or less invasive investigations, and the patient has had persistent symptoms for at least three months or is progressively worsening, an invasive technique such as bronchoscopy with bronchoalveolar lavage (BAL) and lung biopsy for histopathological characteristics are recommended to determine a specific diagnosis [10,27]. The proposed general diagnostic design in chILD is presented in Figure 1 [9,28,29].

### 2.4. Current Principles of Treatment in chILD

Treatment of chILD consists of supportive and pharmacologic therapy that is tailored to different types of disease. Few children do not require any treatment and recover spontaneously. Various drugs (anti-inflammatory, immunosuppressive, anti-fibrotic agents) are used based on data derived from small numbers of patients or studies in adults. However, no guidelines for the treatment of chILD have been proposed so far [2,3,30].

General supportive measures are recommended in all patients. These include adequate nutritional support, oxygen therapy for chronic hypoxemia, bronchodilators for reversible airway obstruction, treatment of intercurrent infections, avoiding exposure to environmental agents, and close attention to childhood immunizations, including annual influenza vaccinations and respiratory syncytial virus prophylaxis [31].

Patients with underlying systemic disorders need specific treatment for the basal disease, such as anti-infective therapy for chronic respiratory infections, chemotherapy for malignancy, management of swallowing dysfunction, and gastroesophageal reflux in patients with chronic aspiration, or avoidance of the offending antigen in HP [31].

If no specific therapy is available, systemic corticosteroids are still the most commonly used drug for selected chILD, considering that suppression of inflammation may be beneficial. A trial of corticosteroids (oral prednisolone or pulse steroid therapy) for >6–8 weeks may be used [31,32]. If glucocorticoids fail, because of significant side effects, or needed for an extended period, alternative immunosuppressive agents such as hydroxychloroquine may been used, which have had anecdotal success [31,33]. Long-term azithromycin treatment has been used for its anti-inflammatory effect in a dose of 10 mg/kg, three days per week [29]. Other immunosuppressive or cytotoxic agents such as azathioprine, cyclophosphamide, cyclosporine, and methotrexate, have also been proposed as an alternative for chILD treatment [31]. Several novel therapeutic strategies are being developed, including substances directed against cytokines, growth factors, and oxidants, which show promising results in some clinical trials in adult patients. IFN-γ (antifibrotic, anti-infective, antiproliferative, and immunomodulatory agent) and pirfenidone (antifibrotic agent) have obtained encouraging results [31,34].

Lung or heart–lung transplantation may be an option for children who have severe and progressive disease and no response to therapy, including highly lethal diseases [31,35,36].

### 2.5. Hypersensitivity Pneumonitis in Children—Diagnosis Approach

Numerous organic (animal- or vegetable-derived proteins, bacteria or fungi) and inorganic (low-molecular-weight chemicals or metals) agents have been implicated in HP. The number of these factors is constantly growing. The most common etiologic agents in children result from pastime activities and consist of avian antigens, such as immunoglobulins, intestinal mucin presented in avian feces, and bloom covering birds’ feathers. They induce a form of HP called pigeon-breeder lung or bird fancier’s disease. Fungi, the causative antigen of classic farmer’s lung disease (Actinomycetes, *Aspergillus* sp., *Penicillium* sp., *Candida* sp., and multiple molds) or inorganic agents (inhaled paints, plastics, and talcum), are rarely reported in this age group. Drugs, such as methotrexate, rituximab, and azathioprine, may also induce HP [5,7,37].

The pathogeny of HP is poorly understood. Complex interactions between nature (type, dimension, and solubility) of allergen, the intensity, duration, and frequency of exposure, and the host immune response in susceptible subjects, are most likely involved. The repetitive or prolonged exposure to environmental inhalant allergens causes type III and type IV hypersensitivity reactions and secondary inflammation of the airways [7].

HP has acute, subacute, and chronic phases, depending on the duration and intensity of exposure to the causative allergen and the patient’s susceptibility. The clinical presentation includes respiratory symptoms such as cough, dyspnea during exercise or rest, tachypnea, and, less frequently, wheezing. Weight loss and fever can also be present. In patients with an acute form of HP, a temporal relation between the respiratory symptoms and antigen exposure can usually be identified. Symptoms mimic a flu-like disease with fever, cough, dyspnea, chest tightness, nausea, and malaise, beginning several hours (4–6) after exposure to the allergen and diminishing over the next 24 h. In cases of subacute or chronic HP, the association between antigen exposure and the development of symptoms may not be evident. Subacute HP develops gradually after prolonged low-dose exposure to the allergen. It is a common presentation of HP in children because of exposure to pet birds at home. Subacute HP with continued exposure to the antigen leads to a chronic form of the disease. Irreversible pulmonary fibrosis, honeycomb lung, and chronic respiratory failure may develop in the absence of exposure interrupting [7,38,39].

As with other forms of chILD, the diagnostic of HP is difficult and challenging in children and depends on integrating the clinical presentation, laboratory tests, imagistic findings, and histologic examination, but no study alone is diagnostic. The key consists of detailed environmental exposure history and, to make a correct diagnosis, the identification of the inciting antigen is essential. Elevated serum immunoglobulins precipitins against the inciting agents are detected in most affected patients [40]. Imagistic investigations are suggestive for the diagnosis of HP. Chest radiograph shows parenchymal opacities and diffuse nodules in acute or subacute HP and persistent reticular opacities in chronic forms. Thoracic HRCT reveals diffuse ground-glass opacities, which are characteristic of acute and subacute HP. Pulmonary fibrosis, secondary parenchymal distortion, bronchiectasis, and progression to a honeycombing aspect may be described in chronic HP [41]. Lung-function tests may usually reveal abnormal pulmonary function with a predominantly restrictive pattern and decreased alveolar diffusion capacity [40]. Analysis of BAL and lung-tissue biopsy may be useful in HP diagnosis. An increase in the total cell count in BAL fluid with marked lymphocytosis is characteristic. Unlike adults, a low CD4+/CD8+ ratio is not a specific feature for children, with an increased value of this ratio being also observed. In dubious cases, a lung biopsy is indicated. The histopathological findings of HP are different, depending on the stage of the disease. Initially, they include diffuse infiltration of interstitial and alveolar spaces with neutrophils, followed by lymphocytes, macrophages, plasma cells, and non-necrotizing granulomas. In chronic HP, interstitial pulmonary fibrosis is the characteristic histopathological lesion [7].

## 3. Case Report

We present the case of a previously apparently healthy 12-year-old boy, who was admitted to our clinic with a three-month history of repeated episodes of dry cough, dyspnea, wheezing, and exercise intolerance. Firstly, symptoms were suggestive of an acute respiratory tract infection (exacerbated cough, dyspnea, and intermittent fever), requiring recurrent hospitalizations. A diagnosis of asthma and bacterial pneumonia were suspected, and different therapeutic regimens were indicated, including oral antibiotics, short cures of oral steroids, antihistamines, antileukotrienes, inhaled bronchodilators, inhaled anti-inflammatory drugs, and antitussive agents, without any clinical improvement. Respiratory symptoms persisted, with periodic exacerbations, without an apparent trigger factor, and weight loss (2 kg) was additionally observed. Three months after the onset of symptoms, the patient was admitted to our clinic. Family history was negative for chronic disorders and he denied any environmental exposure to organic or inorganic agents (avian or animal antigens, fungi, Actinomycetes, *Aspergillus* sp., *Penicillium* sp., *Candida* sp., multiple molds, paints, or plastics). He also denied exposure to any drugs.

Physical examination upon presentation revealed a poor general condition, pallor, fatigue, anorexia, inspiratory dyspnea, tachypnea, and dry cough. The respiratory rate was 30 breaths per minute and oxygen saturation was 90% in room air. The chest exam was significant for bilaterally decreased air movement, wheezing, and diffuse crackles. He was tachycardic, with rhythmic cardiac sounds and normal blood pressure. Digestive, renal, and neurological examinations were normal. No cutaneous, muscular, or articular anomalies were noticed.

**Figure 2:**
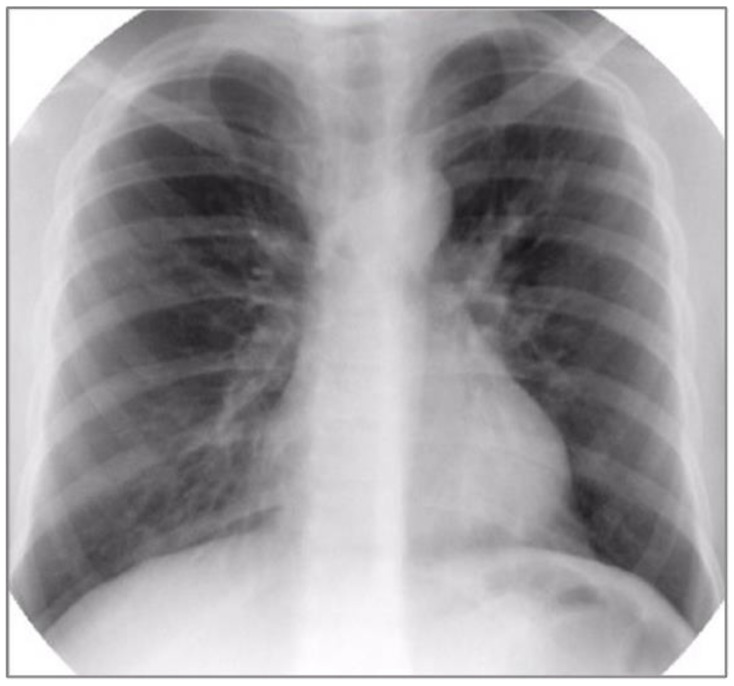
Chest radiography showing diffuse interstitial infiltrates, predominantly perihilar.

**Figure 3:**
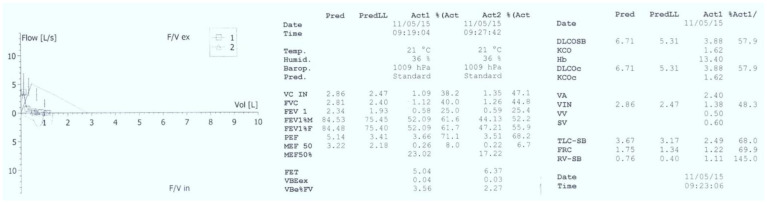
Pulmonary function tests suggestive for severe restrictive and obstructive ventilatory dysfunction and reduced lung capacity for diffusing carbon monoxide.

Basic laboratory investigations revealed hypoxemia (PaO_2_ = 85 mmHg), normocapnia (PaCO_2_ = 35 mmHg), normal complete blood count (CBC), negative inflammatory tests, and normal hepatic and renal function tests. Chest radiography showed diffuse interstitial infiltrates, predominantly perihilar (See Figure 2). Pulmonary function tests were also performed, demonstrating severe restrictive and obstructive ventilatory dysfunction (FVC = 30%, FEV1 = 25%) and reduced lung capacity for diffusing carbon monoxide (DLCO = 3.88 mL/min/mmHg, 57.9% predicted) (See Figure 3).

**Figure 4:**
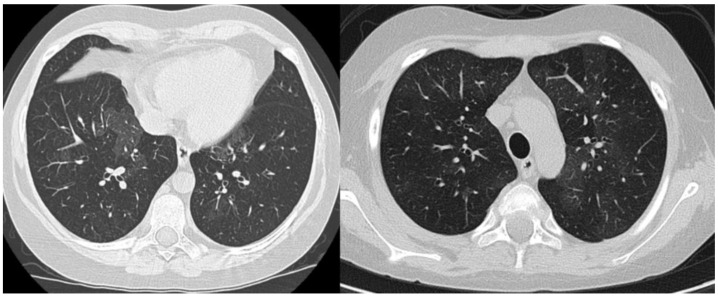
Pulmonary HRCT—transverse sections showing diffuse bilateral ground-glass opacities.

The medical history, with recurrent respiratory signs and symptoms in the absence of an evident trigger and without any extrapulmonary involvement, persistent hypoxemia, and the radiological presentation with diffuse interstitial abnormalities on chest X-ray were major criteria that permitted us to establish the diagnosis of chILD syndrome. Firstly, to alleviate acute manifestations, we initiated treatment with broad-spectrum antibiotics (Ceftriaxone), bronchodilator agents (inhaled Salbutamol), systemic corticosteroids (intravenous Methylprednisolone), oxygen therapy, and respiratory nursing, with slight clinical improvement, relief of dyspnea, and oxygen therapy withdrawal one week after admission. However, pulmonary anomalies on clinical auscultation (bilateral crackles), dry cough and abnormal lung function tests persisted, with minimal alleviation. An HRCT scan of the chest was performed, showing a heterogeneous aspect of lung structure, with diffuse bilateral ground-glass opacities. (See Figure 4).

As part of a differential diagnosis and to establish the etiology of an interstitial lung disease syndrome, we performed additional complex biological analysis. Considering the patient’s age and his unremarkable perinatal history, infancy-specific diseases, such as developmental disorders, alveolar growth abnormalities or surfactant dysfunction disorders, were unlikely. We evaluated the patient’s immune status, with no evidence of tumors on imagistic investigations and negative tests for immunodeficiencies (normal CBC, negative HIV antigen, normal level of serum immunoglobulins and complement, and good response to immunizations). Thus, causes of chILD associated with the immune-compromised host were excluded so we continued work-up for pulmonary disorders of a normal host. Bacterial and fungal cultures, as well as the viral respiratory panel, including SARS-CoV testing, were negative, excluding infectious etiology. Persistent cough, dyspnea, weight loss, and anorexia were suggestive of pulmonary tuberculosis. However, the patient had no history of exposure, and a direct smear examination of sputum along with QuantiFERON-TB Gold evaluation was negative. Considering recurrent respiratory symptoms with tachypnea, hypoxemia, cough, and exercise intolerance, the sweat chloride test and serum alpha 1 antitrypsin level were evaluated, with normal values, excluding cystic fibrosis and alpha 1 antitrypsin deficiency, respectively. A normal cardiovascular system clinical examination and electrocardiogram (ECG), along with a normal echocardiography aspect, excluded a cardiac etiology of the symptoms, as well as pulmonary hypertension. We also performed immunology studies to identify connective-tissue disorders predisposing to diffuse lung disease. Inflammatory markers (erythrocyte sedimentation rate, fibrinogen, and reactive C protein), rheumatoid factor, antinuclear antibody, anti-double-stranded DNA antibody, perinuclear anti-neutrophil cytoplasmic antibody (p-ANCA), cytoplasmic anti-neutrophil cytoplasmic antibody (c-ANCA), complement system measurements (total level, C3 and C4 tests) and circulating immune complexes (CICs) in blood were all negative. Thus, together with the absence of extrapulmonary manifestations, they excluded the autoimmune etiology. HP was also considered, but anamnesis was repeatedly negative for antigen exposure. 

**Figure 5:**
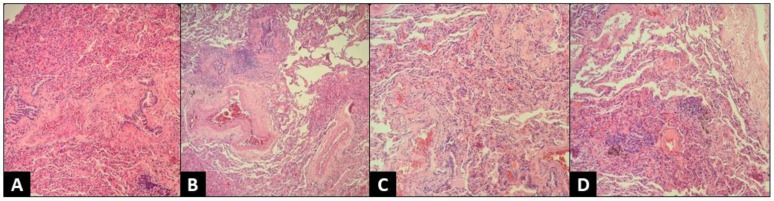
Histopathologic exam—thoracoscopic lung biopsy, H&E stain, 100×. (**A**) Lung parenchyma with bridging peribronchiolar fibrosis; (**B**) Lung parenchyma with non-necrotizing granuloma and peribronchiolar fibrosis; (**C**) Lung parenchyma with interstitial fibrosis and lymphocytic infiltration; (**D**) Lung parenchyma with non-necrotizing granulomas and interstitial fibrosis.

For additional diagnostic purposes, we performed a flexible bronchoscopy, which showed a normal airway anatomy. BAL bacterial and fungal cultures were negative, and cytologic studies excluded alternative causes of chILD such as pulmonary hemorrhage, pulmonary histiocytosis, alveolar proteinosis, and aspiration. BAL cellular analysis revealed lymphocytosis (56%), with an important predominance of CD8+ lymphocytes and a decreased CD4+/CD8+ lymphocytes ratio, strengthening the suspicion of interstitial lung disease. However, considering that, unlike adults, examining BAL fluid can be distinctive but not pathognomonic for certain diseases in children, we decided to perform a lung biopsy using a video-assisted thoracoscopy procedure. Histopathological examination revealed diffuse interstitial and alveolar inflammatory infiltration with numerous lymphocytes, and the absence of neutrophils. Cellular inflammation surrounding bronchioles and reducing alveolar spaces, and reactive type II pneumocyte hyperplasia were present. Lung parenchyma with non-necrotizing granuloma, and peribronchiolar and interstitial fibrosis were also described. Tissue special stains for mycobacteria and fungus were negative. The histopathological aspect of the lung biopsy was characteristic of subacute hypersensitive pneumonitis (Figure 5). 

Considering all these data, we resumed the rigorous history of the patient from all family members regarding a possible contact of the patient with environmental factors, revealing significant exposure to a large quantity of avian antigens (quail and parakeet feathers and droppings), near his home, for one year before the onset of symptoms. To confirm the causative relation between avian antigen exposure, clinical manifestations and imagistic features, we performed lymphocyte transformation testing for quail and parakeets, demonstrating type IV sensitization against quail feathers. We could not measure bird-associated IgG antibodies; this test was not accessible in our clinic. Thus, considering anamnestic, clinical and paraclinical exams, we confirmed the diagnosis of subacute HP secondary to avian antigen (quail) exposure. 

**Figure 6:**
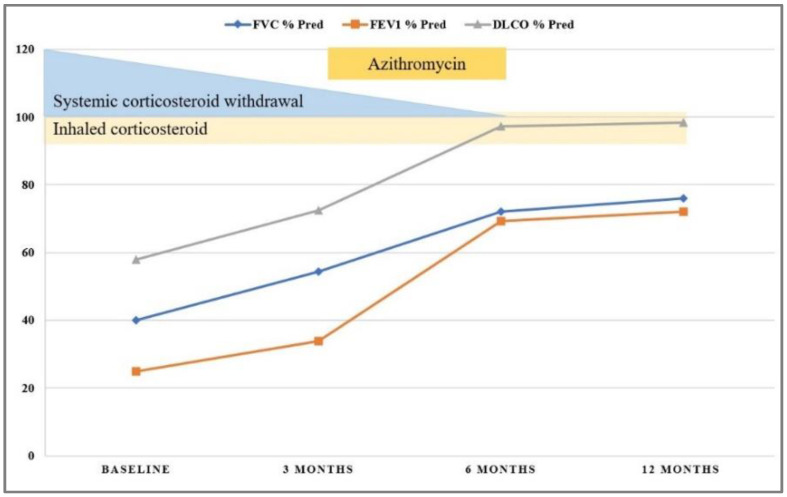
Patient follow-up showing improvement of pulmonary function tests over the first 12 months after admission.

To obtain acute symptomatic relief, we continued systemic corticosteroid therapy using intravenous Methylprednisolone (1 mg/kg/day) for two weeks, followed by a gradual taper to the maintenance dose of 0.5 mg/kg/day of oral prednisolone, which permitted symptom control. High doses of inhaled corticosteroids were added, and the family was advised to eliminate the offending avian antigens from the child’s environment. We established monthly follow-up, and treatment resulted in gradual alleviation of symptoms and slow improvement of pulmonary function. The three months’ follow-up examination revealed normal pulmonary auscultation and cough remission, with the persistence of moderate exercise intolerance. Imagistic evaluation using chest HRCT showed improvement of pulmonary opacities with the resolution of ground-glass attenuation but the presence of some diffuse hypoventilation zones. The treatment was guided by a favorable clinical response, pulmonary function, and radiological improvement, permitting intermittent administration of prednisone. As a second anti-inflammatory line, we added Azithromycin (10 mg/kg/per day, three days per week) for the next three months. Exposure to the causative avian antigen was completely excluded from the patient’s environment, and he remained asymptomatic on the next follow-up visits. Significant improvement of respiratory symptoms and pulmonary function permitted weaning of the oral corticosteroid six months after following initial presentation (See Figure 6). However, low doses of inhalator corticosteroids were continued. Long-term follow-up was favorable, without other acute episodes requiring hospitalization, with symptomatology remission and slow alleviation of imagistic pulmonary aspect, with complete normalization after one year of evolution. 

## 4. Discussion

Hypersensitive pneumonitis is an exceptional interstitial lung disease in children. The diagnosis is difficult and complex investigations are usually needed. Exposure history to an environmental antigen is the key element for diagnosis. A rigorous anamnesis is required in a patient with respiratory symptoms that cannot be explained. Clinical characteristics and routine test abnormalities are suggestive. Still, they are not usually pathognomonic for HP and they often overlap with a multitude of other immune-mediated or infectious lung diseases, as well as cystic fibrosis [42,43]. Radiology and pathology examinations may reveal systemic and lung inflammatory reactions that resemble the reactions in HP and COVID, rather than in other viral pneumonia [44,45]. Specifically, HP should be considered in children with recurrent respiratory symptoms and a history of exposure to environmental inhalator allergens after excluding common etiologies such as infections. Avian antigens are the most common triggers linked to HP [7,46]. However, as in our case, history of exposure is not always possible, complicating the diagnosis approach. 

This paper’s aim is to present the current state of interstitial lung disease in children, regarding specific aspects of diagnostic investigation in this age group, based on a case report of a pediatric patient with histologically confirmed subacute hypersensitive pneumonitis. The peculiarity of this case was the difficulty of establishing the positive diagnosis, the initial anamnesis being nonspecific with denial of exposure to any antigen. Thus, complex paraclinical examinations, including invasive investigations, were necessary. The history of prolonged exposure to avian antigen (quail) was later confirmed by the patient’s parents and the immune response to this trigger was highlighted. 

The initial evaluation included clinical assessment, laboratory testing and imagistic investigations, suggesting interstitial lung disease. Chest HRCT, revealing ground-glass opacities, was suggestive for hypersensitive pneumonitis, but the absence of environmental antigen exposure did not sustain this diagnosis, and more invasive procedures, including flexible bronchoscopy with BAL and lung biopsy, for histopathological analysis were necessary. Particularly in our case, BAL cellular analyses revealed a decreased CD4+/CD8+ lymphocytes ratio, a typical but rare feature of chILD. We made the diagnosis of chILD syndrome, but we could only determine the causative agent after performing lung tissue analysis because of the clinical history that, firstly, was not suggestive of allergen exposure. As in our patient, histopathologic examination of lung biopsy is usually confirmatory, especially when the initial evaluation is not sufficiently convincing of HP. The patient’s family finally revealed anamnestic data of prolonged exposure to avian antigens. Therefore, considering this information, together with recurrent and persistent pulmonary manifestation worsening after antigen exposure, pulmonary auscultation anomalies with bibasilar rales, severe restrictive and obstructive ventilatory dysfunction, compatible changes in chest HRCT, BAL lymphocytosis, and characteristic histopathological features of lung biopsy, the diagnosis of subacute HP secondary to quail antigen exposure was sustained. The positive lymphocyte transformation testing supported HP diagnosis and confirmed the causative relation between avian antigen exposure, clinical manifestations, and imagistic features. 

A difficult anamnesis created a delay in etiology determination so that anatomopathological examination represented the key to diagnosis in our case. However, until the lung biopsy result was available, we performed extensive biological and imagistic work-up since numerous systemic diseases can mimic HP. Having all the clinical, biological and imagistic data suggestive for the diagnostic, we strongly believe that, if the history of chronic exposure to avian antigens had been revealed earlier, we would not have needed to perform a VATS lung biopsy. Thus, the anamnesis revealing chronic exposure to avian antigens, leading to a positive diagnosis, was the key element in this case. 

However, initiating treatment and avian antigen exposure removal in time permitted significant clinical improvement and complete resolution of imagistic abnormalities. The early diagnosis of chILD, including HP, is very important for the therapeutic approach. The timely administration of effective treatment help avoid frequent hospitalizations and prevent progression to irreversible lung damage and pulmonary sequela such as fibrosis, chronic respiratory failure, and cardiac insufficiency.

## 5. Conclusions

Childhood ILD represents a large spectrum of rare and heterogeneous disorders, with important differences compared with adults. Although an increasing proportion of cases can be diagnosed using laboratory, genetic or imagistic investigations, histopathological exam of the lung biopsy still has a significant practical use. While HP is a very rare condition in children, it represents the most frequent interstitial lung disease in this age group. The diagnosis may be challenging because of difficulty identifying triggering factors and overlapping with other forms of ILD. However, it must be considered in any patient with recurrent or prolonged respiratory symptoms, without an obvious etiology. A good prognosis of HP depends on timely recognition and early treatment, preventing progressive pulmonary fibrosis instauration.

## Figures and Tables

**Table 1:** Diagnosis criteria for chILD.

1. Respiratory symptoms (e.g., cough, difficult breathing, exercise intolerance);
2. Respiratory signs (e.g., retractions, tachypnea, clubbing, failure to thrive);
3. Hypoxemia;
4. Diffuse parenchymal abnormalities on chest imaging.

## Data Availability

Not applicable.

## Abbreviations

BAL	bronchoalveolar lavage
CBC	complete blood count
chILD	childhood interstitial lung disease
DLCO	diffusing capacity of the lungs for carbon monoxide
ECG	electrocardiogram
FEV1	Forced Expiratory Pressure in 1 Second
FVC	Forced Vital Capacity
HP	hypersensitivity pneumonitis
HRCT	High-Resolution Computer Tomography
PaCO_2_	Partial Pressure of Carbon Dioxide
PaO_2_	Partial Pressure of Oxygen
X-ray	radiography
